# HSP60 as a Target of Anti-Ergotypic Regulatory T Cells

**DOI:** 10.1371/journal.pone.0004026

**Published:** 2008-12-24

**Authors:** Francisco J. Quintana, Avishai Mimran, Pnina Carmi, Felix Mor, Irun R. Cohen

**Affiliations:** 1 Department of Immunology, The Weizmann Institute of Science, Rehovot, Israel; 2 Center for Neurologic Diseases, Brigham and Women's Hospital, Harvard Medical School, Boston, Massachusetts, United States of America; University of Sheffield, United Kingdom

## Abstract

The 60 kDa heat shock protein (HSP60) has been reported to influence T-cell responses in two ways: as a ligand of toll-like receptor 2 signalling and as an antigen. Here we describe a new mechanism of T-cell immuno-regulation focused on HSP60: HSP60 is up-regulated and presented by activated T cells (HSP60 is an ergotope) to regulatory (anti-ergotypic) T cells. Presentation of HSP60 by activated T cells was found to be MHC-restricted and dependent on accessory molecules - CD28, CD80 and CD86. Anti-ergotypic T cells responded to T-cell HSP60 by proliferation and secreted IFNγ and TGFβ1. *In vitro*, the anti-ergotypic T cells inhibited IFNγ production by their activated T-cell targets. *In vivo*, adoptive transfer of an anti-ergotypic HSP60-specific T-cell line led to decreased secretion of IFNγ by arthritogenic T cells and ameliorated adjuvant arthritis (AA). Thus, the presentation of HSP60 by activated T cells turns them into targets for anti-ergotypic regulatory T cells specific for HSP60. However, the direct interaction between the anti-ergotypic T regulators (anti-HSP60) and the activated T cells also down-regulated the regulators. Thus, by functioning as an ergotope, HSP60 can control both the effector T cells and the regulatory HSP60-specific T cells that control them.

## Introduction

The 60 kDa HSP molecule (HSP60) has been found to induce the down-regulation of immune inflammation. Vaccination with self-HSP60 or its peptide epitopes can arrest the development of type 1 diabetes (T1DM), spontaneous or induced, in non-obese diabetic (NOD) mice [1], [2], [3] and in other mouse models [4]; HSP60 vaccination can also inhibit adjuvant arthritis (AA) in rats [5], [6], [7]. In humans, spontaneous HSP60-specific T-cell responses are associated with a relatively good prognosis in juvenile rheumatoid arthritis [8], [9], probably due to the activity of HSP60-specific regulatory cells [10], [11]. HSP60-based immuno-regulation is being exploited clinically for the treatment of autoimmune disorders; the administration of a peptide from human HSP60 has been reported to halt β-cell destruction in newly diagnosed T1DM patients [12], [13] and to halt experimental Sjögren's syndrome [14]. An HSP60 peptide is also being developed to treat human rheumatoid arthritis [7]. Thus is important to characterize the immune regulatory mechanisms triggered by HSP60.

HSP60 appears to affect immune inflammation by at least two different mechanisms: as a ligand for innate immune receptors and as an antigen recognized by adaptive immune receptors.

HSP60 can control T cells by innate receptor dependent mechanisms. Soluble human HSP60 was found to directly signal human T cells via toll-like receptor 2 (TLR2). The activation of TLR2 dependant signalling on effector T cells has two consequences: First, it inhibits the migration of T cells towards the chemokine SDF-1 – irrespective of their antigen-specificity – and so inhibits the general entry of T cells into inflammatory sites [15]. Second, it down-regulates T-bet, NF-κB, and NFAT, and up-regulates GATA-3, leading to decreased secretion of TNFα and IFNγ and enhanced secretion of IL-10 by the responding T cells [16]. In addition to its direct effects on effector T cells, HSP60-triggered activation of TLR2 signalling boosts the suppressive activity of human CD4^+^CD25^high^ T cells [17]. These innate, TLR-2 mediated effects of soluble HSP60 can down-regulate T-cell dependent inflammation.

HSP60 can also control T cells by T-cell receptor dependent mechanisms. In T1DM, for example, vaccination with HSP60 epitopes activates HSP60-specific regulatory T cells that affect the T-cell response to disease-associated antigens (insulin, glutamic acid decarboxylase and HSP60 itself) by inducing a shift from the secretion of INFγ to IL-10 [3], [18]; HSP60 vaccination induces a similar cytokine shift in the response to the mycobacterial antigens that trigger AA [6], [7]. It has been proposed that HSP60-specific regulatory T cells respond to HSP60 expressed and presented by stressed tissue cells at sites of inflammation [19]. According to this view, down-regulation of tissue inflammation is mediated through a by-stander effect – the Th2 cytokines produced at the site by the HSP60-specific T cells down-regulate the Th1 response of adjacent effector T cells reacting to other tissue antigens [20], [21].

We now report an additional mechanism by which HSP60 down-regulates inflammation: activated effector T cells up-regulate HSP60 and present their own HSP60 epitopes to anti-ergotypic regulatory T cells. Anti-ergotypic regulatory T cells recognize peptides derived from molecules – ergotopes – expressed by T cells primarily when they are activated [22], [23]. Thus far, several ergotopes have been defined: the CD25 molecule and the TNFα receptor are examples [24], [25]. Anti-ergotypic T cells, detectable in humans [26], [27] and in experimental animals [23], are heterogeneous: they include CD4^+^ and CD8^+^ T-cell receptor αβ (TCRαβ) and TCRγδ T cells [26], [27], [28], [29]. Most importantly, anti-ergotypic T cells have been shown to down-regulate damaging autoimmune inflammation in the model autoimmune diseases experimental autoimmune encephalomyelitis (EAE) [23], [25] and AA [24].

Here we show that HSP60 fulfils the three defining properties of a T-cell ergotope: activated T cells express HSP60; HSP60 epitopes are presented by T-cell MHC molecules and trigger specific anti-ergotypic T cells; and the HSP60-specific anti-ergotypic T cells can down-regulate pro-inflammatory effects of effector T cells *in vitro* and *in vivo*. In other words, HSP60 is an immune regulatory signal produced not only by stressed tissues, but also by activated effector T cells in need of immune regulation.

## Results

### DNA vaccination with pHSP60 activates anti-ergotypic responses

**Figure 1:**
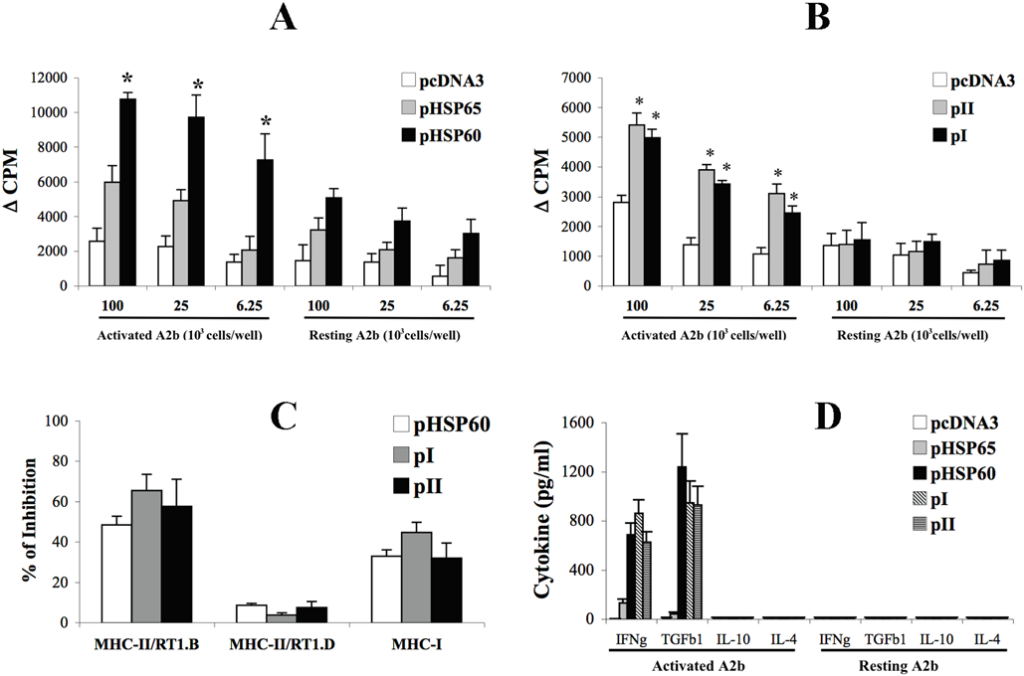
DNA vaccination with HSP60 induces anti-ergotypic T cells. A and B. Anti-ergotypic proliferative response of LNC from rats vaccinated with pcDNA3, pHSP65 or pHSP60 (A) or pcDNA3, pI or pII (B), taken 26 days after the induction of AA. Proliferative responses are presented as the ΔCPM±SEM of quadruplicate cultures. * p<0.05 compared to the pHSP65 (A) or the pcDNA3 (B) groups. C. Monoclonal antibodies to MHC-II/RT1.B, MHC-II/RT1.D or MHC-I were assayed for their ability to block the anti-ergotypic proliferative response. Results are presented as the percent of inhibition of proliferation±SEM of quadriplicate cultures. D. Anti-ergotypic cytokine response of LNC taken from rats vaccinated with pcDNA3, pHSP65, pHSP60, pI or pII 26 days after the induction of AA. IFNγ (IFNg), TGFβ1 (TGFb1), IL-10 and IL-4 were quantified in the culture supernatants after 72 hr of stimulation with 10^5^ activated or resting, irradiated, A2b cells per well. The results are presented as pg/ml±SEM of triplicate cultures. Three independent experiments produced similar results.

We have reported that DNA vaccination with the *hsp60* gene (pHSP60) or with its N-terminal fragments – constructs pI (aa 1–130) or pII (aa 120–240)– induced HSP60-specific T cells and inhibited the development of AA [6], [7]. DNA vaccination with mycobacterial HSP65 (pHSP65) also protected rats against AA [30], but this vaccination was significantly less effective than was vaccination with self-HSP60 [6]. Does protective HSP60 vaccination activate anti-ergotypic reactivity? To approach this question, we studied the anti-ergotypic T-cell responses in rats vaccinated with pHSP60, pI, pII, pHSP65 or pcDNA3, 26 days after the induction of AA. Lymph node cells (LNC) of the vaccinated rats were incubated with irradiated activated or resting A2b T cells, and proliferative responses were measured to different numbers of A2b stimulator cells. Figure 1A shows that vaccination with pHSP60 induced a proliferative anti-ergotypic T-cell response, which was significantly (p<0.05) higher than that induced by pHSP65. Moreover, vaccination with the pI or pII constructs of HSP60 also induced a significant (p<0.05) anti-ergotypic response compared to pcDNA3 (Figure 1B). Using neutralizing antibodies, we found that the anti-ergotypic response induced by DNA vaccination included both MHC-II (RT1.B) and MHC-I restricted T cells (Figure 1C).

Note that the pHSP60 DNA vaccine also increased the response to resting A2b T cells, but to a lower extent than to activated A2b T cells (Figure 1A). However, only activated A2b T cells induced cytokine secretion – characterized by secretion of IFNγ and TGFβ1, but not of IL-10 or IL-4 (Figure 1D).

### Peptide Hu3 of HSP60 activates an anti-ergotypic response

**Figure 2:**
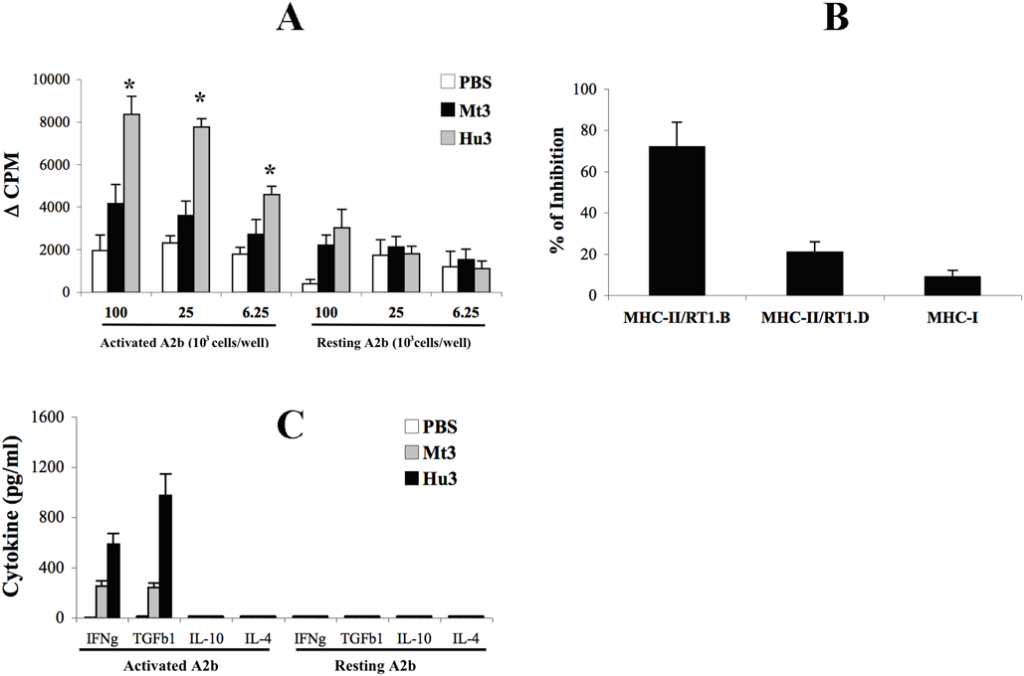
Vaccination with HSP60 peptide Hu3 induces anti-ergotypic T cells. A. Anti-ergotypic proliferative response of LNC from rats vaccinated with PBS, Mt3 or Hu3 in IFA, taken 26 days after AA induction. Proliferative responses are presented as the ΔCPM±SEM of quadruplicate cultures. * p<0.05 compared to the Mt3 group. B. Monoclonal antibodies to MHC-II/RT1.B, MHC-II/RT1.D or MHC-I were assayed for their ability to block the anti-ergotypic proliferative response. Results are presented as the percent of inhibition of proliferation±SEM of quadruplicate cultures. C. Anti-ergotypic cytokine response of LNC taken from rats vaccinated with PBS, Mt3 or Hu3 in IFA, 26 days after AA induction. IFNγ (IFNg), TGFβ1 (TGFb1), IL-10 and IL-4 were quantified in the culture supernatants after 72 hr of stimulation with 10^5^ activated or resting, irradiated, A2b cells per well. The results are presented as pg/ml±SEM of triplicate cultures. Three independent experiments produced similar results.

Vaccination with the HSP60 peptide Hu3 (aa 31–50) can also inhibit AA [7]. Does effective HSP60-peptide vaccination also induce an anti-ergotypic response? Figure 2A shows that vaccination with peptide Hu3 was significantly (p<0.05) more effective in inducing an anti-ergotypic proliferative response than was vaccination with the homologous, immunogenic Mt3 peptide from mycobacterial HSP65. The anti-ergotypic proliferative response induced by peptide Hu3 was also more focused in its MHC-II restriction (Figure 2B); recall that HSP60 DNA vaccination led to anti-ergotypic proliferative responses that included both MHC-I and MHC-II restricted T cells (Figure 1C). The anti-ergotypic T cells induced by Hu3 peptide vaccination secreted IFNγ and TGFβ1, but not IL-10 or IL-4 in response to activated A2b T cells (Figure 2C).

### T-cell activation up-regulates HSP60 expression

**Figure 3:**
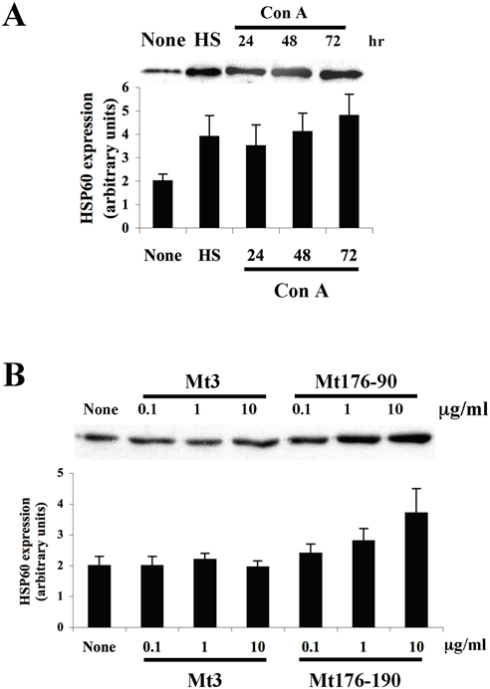
T-cell activation up-regulates cellular levels of HSP60. A. LNC were stimulated with Con A for 24, 48 or 72 hr, subjected to a 30 minutes 42°C heat shock (HS) or kept at 37°C (None). Cell lysates were prepared and HSP60 expression was analyzed by western blot with specific antibodies, and quantified (in arbitrary units). B. A2b T-cells were stimulated with various concentrations of the target peptide Mt176-90, a control peptide (Mt3) for 72 hr, or with medium alone (None). Cell lysates were prepared and HSP60 expression was analyzed by western blot with specific antibodies, and quantified (in arbitrary units). Two independent experiments produced similar results.

The above results (Figures 1 and 2) suggested that the inhibition of AA by HSP60 DNA or peptide vaccination was associated to the induction of anti-ergotypic proliferative and cytokine responses to activated, syngeneic T cells; but do epitopes of HSP60 function as ergotopes? Is HSP60 up-regulated and presented on activated T cells to anti-ergotypic T cells? To study this question, we compared the expression of HSP60 in activated or resting T cells by western blot. LNC were incubated for 1, 2 or 3 days with the T-cell mitogen Con A, or left untreated. Cell lysates were prepared at the end of the incubation, standardized by protein content, and analyzed by western blot for the expression of HSP60. As a positive control for the induction of HSP60, LNC were also heat shocked for 30 minutes at 42°C and allowed to recover for 4 hr at 37°C. Figure 3A shows that T-cell activation with Con A or heat shock triggered a similar increase in the expression levels of HSP60. No differences in total protein content were seen when the different samples were analyzed by PAGE-SDS (data not shown).

We also detected up-regulation of HSP60 following activation of the T-cell clone A2b by its target peptide epitope Mt176-90 but not by the control peptide Mt3 (Figure 3B); no differences in total protein were seen when the samples were analyzed by PAGE-SDS (data not shown). The up-regulation of HSP60 protein is in agreement with previous studies done at the level of mRNA expression [31], and demonstrates that T-cell activation by specific antigen leads to the up-regulation of cellular HSP60.

### Activated T cells stimulate HSP60-specific T cells

**Figure 4:**
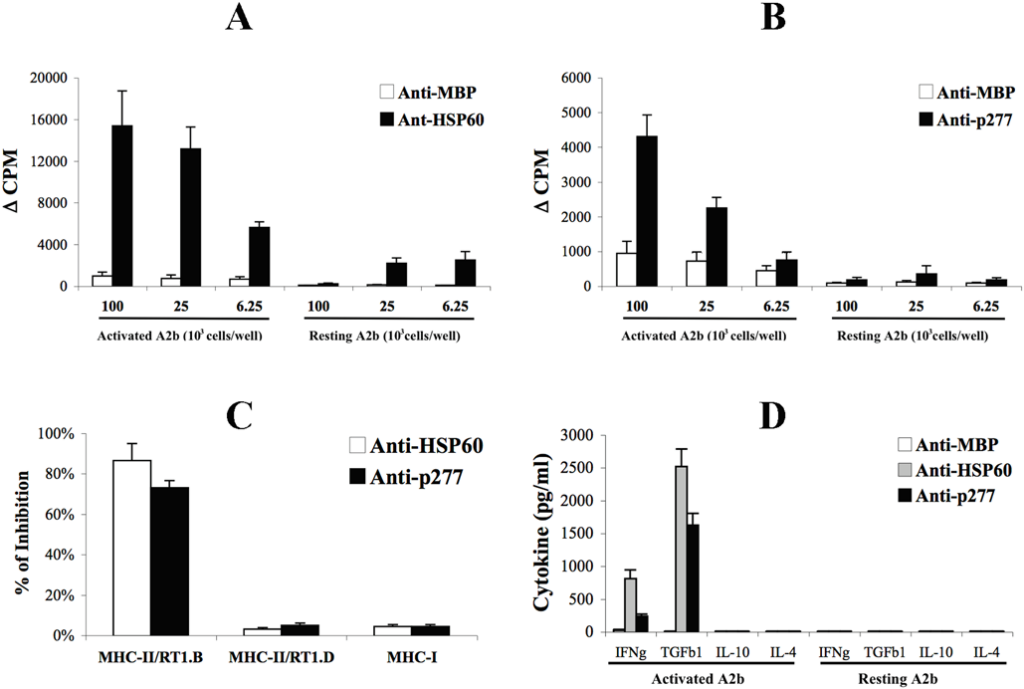
MHC class II-restricted recognition of activated T cells by HSP60-specific T-cells. A. Anti-ergotypic proliferative response of Anti-HSP60 or Anti-MBP T cell lines. Proliferative responses are presented as the ΔCPM±SEM of quadruplicate cultures. B. Anti-ergotypic proliferative response of Anti-p277 or Anti-MBP T cell lines. Proliferative responses are presented as the ΔCPM±SEM of quadruplicate cultures. C. Monoclonal antibodies to MHC-II/RT1.B, MHC-II/RT1.D or MHC-I were assayed for their ability to block the anti-ergotypic proliferative response of the Anti-HSP60 and the Anti-p277 T cell lines. Results are presented as the percent of inhibition of proliferation±SEM of quadruplicate cultures. D. IFNγ (IFNg), TGFβ1 (TGFb1), IL-10 and IL-4 were quantified in the culture supernatants after 72 hr of stimulation of the Anti-MBP, Anti-p277 or Anti-HSP60 T cell lines with 10^5^ activated or resting, irradiated, A2b cells per well. The results are presented as pg/ml±SEM of triplicate cultures. Three to five independent experiments produced similar results.

Are HSP60 epitopes actually presented by activated T cells? We studied this question using HSP60-specific T-cell lines as probes for HSP60-epitope presentation, and a control T-cell line specific for MBP. Activated or resting A2b T cells were irradiated to inhibit their proliferation, and their presentation of HSP60 epitopes was probed with the test T-cell lines. Figure 4A shows that the Anti-HSP60 T cells proliferated upon incubation with activated A2b; the response to resting A2b T-cells was significantly lower. The reaction to HSP60 was specific; the Anti-MBP T cells failed to respond to the A2b T cells, irrespective of their state of activation. Thus, only the activated A2b T cells presented HSP60 epitopes recognizable by the Anti-HSP60 line. The proliferation of the Anti-HSP60 line was restricted through the MHC-II/RT1.B molecule (Figure 4C).

The 437-60 region of HSP60 (contained in the HSP60 peptide designated p277) is an immunodominant T-cell epitope in the Lewis rat [32]. We could therefore use an Anti-p277 T-cell line to investigate whether activated A2b T cells presented the defined HSP60 peptide epitope p277. Although less than the Anti-HSP60 T-cell line (compare Figures 4A and 4B), the Anti-p277 T cells showed a significant proliferation upon incubation with activated A2b T cells (Figure 4B), but not with resting A2b T cells. This anti-ergotypic proliferative response was MHC-II/RT1.B restricted (Figure 4C). Thus, activated T cells can present a specific epitope of their up-regulated HSP60 molecules – the p277 peptide epitope; that this occurs in the absence of any other APC, suggests that activated T cells can process and present epitopes of their own HSP60. Thus, T-cell presentation of HSP60 can reveal the state of activity of a T cell.

To investigate how the anti-ergotypic response to HSP60 might function, we analyzed the cytokines produced by Anti-HSP60 and Anti-p277 T cells in response to either activated or resting A2b T cells. Figure 4D shows that both the Anti-HSP60 and Anti-p277 T-cells secreted relatively small amounts of IFNγ and relatively high amounts of TGFβ1 upon stimulation with activated A2b T cells only. The T cells did not secrete IL-10 or IL-4.

### The activation of HSP60-specific anti-ergotypic T cells requires co-stimulation

**Figure 5:**
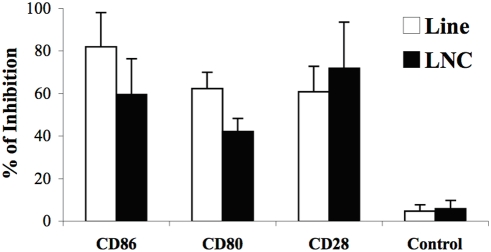
The activation HSP60-specific anti-ergotypic T cells requires co-stimulation. Monoclonal antibodies to CD28, CD80 or CD86, or a control IgG (Control), were assayed for their ability to block the anti-ergotypic proliferative response of Anti-HSP60 T-cells (Line) or of LNC prepared from pHSP60-vaccinated rats (LNC). Results are presented as the percent of inhibition of proliferation±SEM of quadruplicate cultures. Three independent experiments produced similar results.

Complete T-cell activation is achieved when TCR-mediated signaling is reinforced by signals originating from co-stimulatory molecules such as CD28. CD28 interacts with CD80 and CD86 molecules displayed on the surface of APC [33]. Activated T cells [34], [35] and activated A2b [28] express CD80 and CD86 molecules on their surface. We therefore studied the need for CD80, CD86 and CD28 in the activation of HSP60-specific T cells by activated T cells. LNC prepared from pHSP60 vaccinated rats, or Anti-HSP60 T cells, were stimulated with irradiated A2b T cells in the presence of blocking antibodies to CD80, CD86 or CD28. Figure 5 shows that incubation with each one of these antibodies produced a significant inhibition in the anti-ergotypic response of HSP60-specific T cells. Hence, co-stimulation by way of CD80, CD86 and CD28 appears to be required for the activation of anti-ergotypic HSP60-specific T cells by activated T cells.

### HSP60-specific anti-ergotypic T cells ameliorate AA

**Figure 6:**
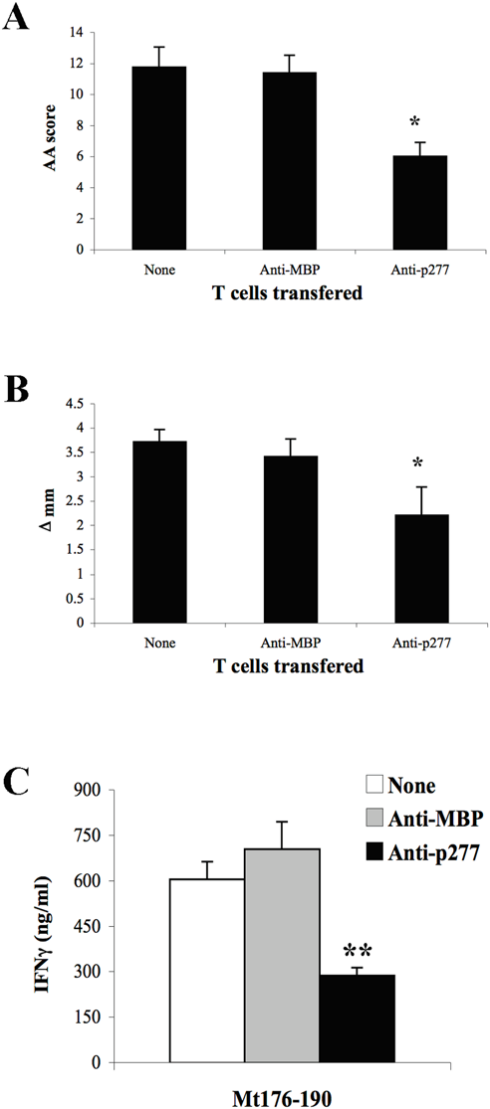
HSP60-specific anti-ergotypic T-cells control arthritogenic T-cells *in vivo*. A and B. Anti-MBP or Anti-p277 T cells were injected ip into naïve Lewis rats and three days later AA was induced. Twenty-six days after AA induction, at the peak of AA, the AA clinical score (A) and the hind paw diameter (B) were determined. The bars represent the mean values ± SEM for each group of 8 rats. C. LNC were collected on day 26 after AA induction and the secretion of IFNγ upon stimulation with Mt176-90 was studied. The results are presented as pg/ml±SEM of triplicate cultures. Three independent experiments produced similar results. * p<0.05 and ** p<0.005 compared to the Anti-MBP group.

If HSP60-specific anti-ergotypic T cells are indeed regulatory, then it should be possible to inhibit inflammatory disease by adoptively transferring them. We tested the effects of Anti-p277 T cells on AA by transferring 10^7^ cells to rats 3 days before the active induction of AA. As a control, we used the Anti-MBP T-cell line. The rats were scored for signs of arthritis, and the hind paw diameter was measured with a caliper on day 26, the peak of AA [36]. Figure 6 shows that the recipients of the Anti-p277 cells showed a significant reduction in the signs of AA, both in terms of arthritis score and of limb swelling. The Anti-MBP T cells had no effect on the progression of AA (Figure 6).

**Figure 7:**
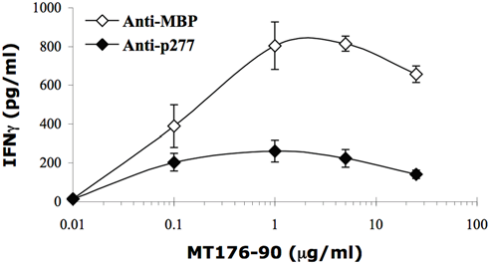
HSP60-specific Anti-ergotypic T-cells control arthritogenic T-cells *in vitro*. LNC from Mt immunized rats (2.5×10^5^ per well) were activated with Mt176-90 for 72 hr in the presence of Anti-p277 or Anti-MBP T-cells (5×10^4^ per well). The secretion of IFNγ was determined by ELISA, the results are presented as pg/ml±SEM of triplicate cultures. The differences between the groups were significant (p<0.05) for antigen concentrations higher than 0.1 µg/ml. Three independent experiments produced similar results.

The arthritogenic T cells that drive AA have a Th1 phenotype. Accordingly, lymph node T cells from rats suffering from AA rats secrete high levels of IFNγ in response to *in vitro* stimulation with the Mt176-90 peptide [6], containing the pathogenic 180-88 T-cell epitope of the mycobacterial 65 kDa HSP [37]. The inhibition of AA achieved by vaccination with HSP60 or its peptides is reported to be associated with a reduction in INFγ production induced by Mt176-90 [6], [7]. We therefore isolated LNC from rats adoptively transferred with Anti-p277 or Anti-MBP T cells, and studied the secretion of IFNγ upon stimulation with Mt176-90. Figure 7C shows that the transfer of Anti-p277 T cells led to a significant reduction in the secretion of IFNγ in response to the AA target peptide Mt176-190. Thus, HSP60-specific T cells, demonstrating anti-ergotype activity, down-regulate IFNγ secretion by the candidate pathogenic T cells at the time they adoptively down-regulate AA.

### HSP60-specific anti-ergotypic T cells modulate effector T-cell IFNγ in vitro

The results obtained in the AA model demonstrated that anti-ergotypic HSP60-specific T-cells can control effector T cells by adoptive transfer *in vivo*. To further investigate the effect of HSP60-specific anti-ergotypic T cells, we tested whether these T cells might be able to directly regulate *in vitro* the IFNγ secretion of LNC taken from rats on day 26, at the peak of AA. LNC of rats with actively induced AA were prepared and activated with Mt176-90 in the presence of the Anti-p277 anti-ergotypic T-cell line, or in the presence of the control Anti-MBP T-cell line. T cells reactive with Mt176-90 have been shown to transfer AA to irradiated naïve Lewis rats [38]. Co-incubation with the Anti-p277 line, but not with the Anti-MBP line, led to a significant decrease in the secretion of IFNγ (Figure 7). We did not detect a concomitant induction of IL-10 (not shown). Thus, anti-ergotypic T cells can directly control *in vitro* the arthritogenic T-cell IFNγ cytokine response.

### HSP60-specific regulators become anergic while regulating activated T cells

**Figure 8:**
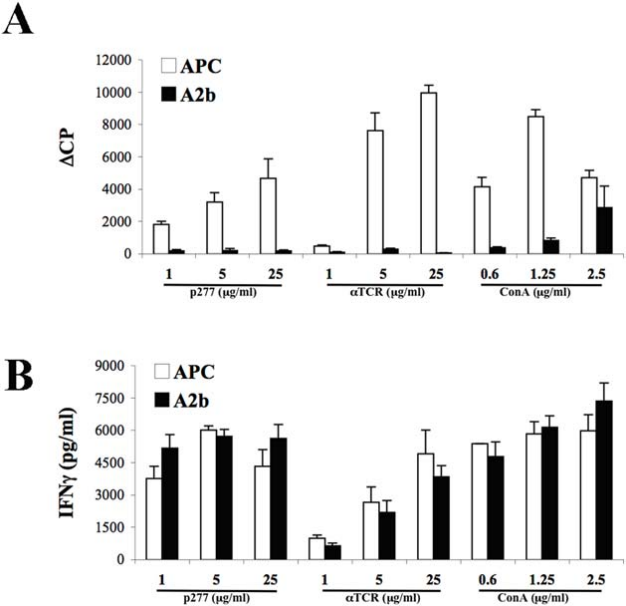
Anti-ergotypic HSP60-specific T cells become anergic after interacting with activated T cells. Anti-ergotypic Anti-p277 T cells were stimulated for 3 days with irradiated, activated A2b cells (A2b) or with irradiated APC fed with the p277 peptide (APC). The Anti-p277 T cells were maintained for 4 additional days in culture, and stimulated with APC and p277 peptide, Con A or immobilized anti-TCR (αTCR) antibodies. T-cell proliferation (A) and IFNγ (B) release were measured after 3 days. The proliferative responses are presented as the ΔCPM (±SEM) (A), and the IFNγ as pg/ml±SEM (B) of triplicate cultures. Three to five independent experiments produced similar results.

We have shown in the previous sections that HSP60-specific anti ergotypic T cells can recognize and down-regulate arthritogenic T cells, *in vitro* and *in vivo*. However, any regulatory mechanism has to be regulated; uncontrolled down-regulation of immunity would be as detrimental to the organism as uncontrolled autoimmunity [22]. We therefore studied whether the stimulation of HSP60-specific anti-erogotypic T cells by activated T cells might itself affect the regulators. In other words, might the anti-ergotypic HSP60-specific T-cell lines be affected differently by seeing their HSP60 epitopes presented by activated T cells compared to recognizing HSP60 presented by classical APC? To study this possibility, we incubated the Anti-p277 line for 3 days with either irradiated APC and p277 peptide or with irradiated, activated A2b T cells. The Anti-p277 T cells were then recovered from the cultures, maintained for 4 additional days in culture without APC or A2b T cells, and then stimulated with APC and p277 peptide, with mitogenic Con A or immobilized anti-TCR. Figure 8 presents the outcome. It can be seen that the Anti-p277 T cells, following co-culture with APC and p277 could still respond to a second stimulation with APC and p277 or with mitogenic αTCR or ConA; however, previously co-culturing the anti-p277 line with activated A2b T cells rendered the Anti-p277 line anergic; the line now failed to proliferate in response to APC and p277 or to either of the two mitogens (Figure 8A). The Anti-p277 T cells could still secrete IFNγ (Figure 8B) but not TGFβ1, IL-10 or IL-4, despite their failure to proliferate. The Anti-p277 line cells, however, went on to die *in vitro* after their exposure to the activated A2b T cells. Thus, it appears that the interaction of anti-ergotypic T-cell lines with their target activated effector T cells leads to anergy and loss of the anti-ergotypic T cells; activated effector T cells and regulator T cells can down-regulate each other. In contrast, anti-ergotypic T cells can be maintained in culture by APC and specific peptide antigen [22].

## Discussion

The present study extends the role of HSP60 in immune regulation; HSP60, as we show here, can also function in anti-ergotypic T-cell regulation. Anti-ergotypic T cells have been identified as a population of regulatory T cells expanded by T-cell vaccination regimes [22], [28]. T cell vaccination has shown promising results in clinical trials that studies its potential as a therapy for multiple sclerosis and rheumatoid arthritis [39], [40], [41]. Notably, T cell vaccination induces two types of anti-ergotypic regulatory T cells: CD4^+^CD25^high^Foxp3^+^ regulatory T cells and also CD4^+^CD25^high^Foxp3^−^ regulatory T cells [39], [40]. The anti-ergotypic T cells reactive with HSP60 described in this manuscript did not express the transcription factor Foxp3 (data not shown) and are therefore similar to the CD4^+^CD25^high^Foxp3^−^ T cells described by Zhang and coworkers [39], [40]. The association of these anti-ergotypic regulatory cells with a beneficial effect of T cell vaccination on multiple sclerosis and rheumatoid arthritis [39], [40], [41], demonstrates the importance of this regulatory cell population as a target for human immunotherapy. The results presented here demonstrate that HSP60 is an ergotope, shedding light on two separate but overlapping issues: the various roles of HSP60 in immune regulation and the nature of anti-ergotypic T regulators.

Vaccination with HSP60 DNA or peptide, previously shown to down-regulate AA [6], [7], can activate various types of anti-ergotypic responses (Figures 1 and 2). Similarly to what we observed for DNA vaccination with the CD25 ergotope [28], DNA vaccination with HSP60 or its fragments induced both MHC-I and MHC-II restricted anti-ergotypic T cells (Figure 1). Hu3 peptide vaccination, conversely, only activated an MHC-II restricted response (Figure 2). Therefore we can conclude that activated T cells process their endogenous HSP60 by both the MHC-I and the MHC-II pathways of antigen presentation. Moreover, our results suggest that the anti-ergotypic T-cell population responsive to HSP60 is heterogeneous, and different methods of HSP60 vaccination (DNA or peptides) might be used to expand particular HSP60-specific anti-ergotypic subpopulations.

It is intriguing that ergotopes such as HSP60 and CD25 are presented by MHC-II molecules. Peptides derived from endogenous antigens are usually MHC-I restricted, while MHC-II molecules mainly present peptides provided by the endocytic pathway of antigen processing [42]. The processing and presentation of endogenous antigens via by the MHC-II pathway has been extensively reported and is thought to result from active mechanisms of autophagy operating in T cells [43], [44]. However, the existence of natural anti-ergotypic regulatory T cells reactive with HSP60 differentiates the MHC presentation of an ergotope from the presentation of other self-antigens: activation-triggered expression of HSP60 turns T cells into targets of HSP60-specific anti-ergotypic regulation. It remains to be seen whether HSP60 epitopes are also presented by other, non-classical MHC molecules. Q1a, for example, is a non-classical MHC-I molecule expressed on T cells that can present endogenous epitopes to regulatory T cells of the CD8^+^ type [45]. These Q1a-mediated interactions have been shown to control EAE through a T-cell network that involves both CD4^+^ and CD8^+^ T cells [46], [47]; it is possible that the Q1a regulatory network also includes anti-ergotypic T cells reactive to HSP60.

HSP60 is also a target of natural T and B cell autoreactivity. Natural antibodies to HSP60 are detectable in the serum of healthy humans [48], [49] and mice [50]; the IgG isotype of these antibodies reveals the activation of helper HSP60-specific T cells. Actually, human cord blood contains a high frequency of T cells responsive to HSP60, and repertoires of healthy humans contain T cells reactive to self-HSP60 [51]. The prevalence of autoimmunity to HSP60 suggests that it serves as a component of the immunological homunculus [52], [53], and mediates an anti-inflammatory effect [19]. Indeed, T-cell reactivity to HSP60 is associated with a good prognosis in juvenile arthritis [8], [54], and the administration of a peptide of HSP60 to newly diagnosed patients with T1DM can arrest the autoimmune destruction of β-cells and induce a Th1 to Th2 shift in the diabetogenic T cells [13]. Autoimmunity to HSP60, naturally acquired or induced by vaccination, bodes well for healthy immune regulation.

How might HSP60-specific anti-ergotypic T cells control the arthritogenic T cells? The anti-inflammatory properties of HSP60-specific T cells are thought to rely on a local by-stander effect exerted on other T cells via regulatory cytokines such as IL-10 and TGFβ1 [55], [56]. We have found (Figures 1, 2 and 4), that the HSP60-specific anti-ergotypic T cells secrete TGFβ1, a cytokine with immuno-modulatory functions [55]. Indeed, the HSP60-specific anti-ergotypic T cells controlled the activity of arthritogenic T cells both *in vivo* (Figure 6) and *in vitro* (Figure 7). We propose that upon recognition of their target HSP60 epitopes within MHC molecules on the surface of activated T cells, the anti-ergotypic T cells can modulate the pro-inflammatory activity of effector T cells via the secretion of TGFβ1, and perhaps other, yet undetected regulatory cytokines. Note that upon transfer of anti-ergotypic HSP60-specific T cells, we detected a decrease in the activity of the arthritogenic T cells that secrete IFNγ (Figure 6). However, we did not detect a concomitant increase in the number of IL-10 secretors, as we have previously described upon vaccination with DNA vaccines coding for HSP60 or its fragments [6], [7], or following vaccination with the HSP60 peptide Hu3 [7]. Hence, the participation of HSP60 in anti-ergotypic regulatory networks might only account for some, but not all, of the regulatory activities of HSP60-specific T cells. Indeed, it has been recently shown that HSP60 can have direct anti-inflammatory effects on human T cells which are TLR2-dependant: free HSP60 can inhibit T-cell migration towards the inflammatory chemokine SDF-1A [15] and modify the levels of transcription factors involved in T-cell polarization, leading to decreased secretion of TNFα and IFNγ and enhanced secretion of IL-10 [16], [17].

Finally, the present study indicates that HSP60-specific anti-ergotypic regulatory T cells can be down-regulated as a result of their interaction with their target T cells (Figure 8). Indeed, the induction of a strongly arthritogenic T-cell effector response leading to adjuvant arthritis was found to suppress the anti-ergotypic regulator response; this down-regulation of the regulators by the effectors could be overcome by vaccinating the rats with an ergotope, CD25 [24]. Thus, the magnitude of an immune response can be influenced decisively by a dynamic balance between regulators and effectors [28]. In this light, we can view the complex roles of HSP60 in immuno-regulation: HSP60 facilitates the recognition and control of activated T cells via anti-ergotypic interactions, and this regulatory interaction also down-regulates the activity of the HSP60-specific regulators. The regulatory molecules on activated T cells that down-regulate the anti-ergotypic regulators remain to be discovered; however, it has been reported that mutual interactions between T cells (T-T interactions) can induce T-cell anergy [57], [58]. Thus, the numbers of regulatory T cells reactive to HSP60 might be positively influenced by the presentation of HSP60 by professional APC and negatively influenced by the presentation of HSP60 by activated T cells [59]; regulators, too, need regulation [22].

In conclusion, our results show that the varied regulatory mechanisms mediated by HSP60 are complex indeed; not only does the immune system use innate and adaptive receptors to respond to HSP60 expressed *in situ* by the tissues and in body fluids [60], we can now conclude that the immune system uses its own HSP60 expressed on activated T cells as a marker that reflects their functional state and their need of regulation.

## Materials and Methods

### Rats

Female Lewis rats were raised and maintained under pathogen-free conditions in the Animal Breeding Center of the Weizmann Institute of Science. Experiments were carried out under the supervision and guidelines of the Animal Welfare Committee. The rats were 1–2 months old at the start of the experiments.

### Antigens, peptides, antibodies and adjuvants

*M. tuberculosis* (Mt) strain H37Ra was obtained from Difco (Detroit, MI, USA). Mt purified protein derivative (PPD) was provided by the Statens Seruminstitut (Copenhagen, Denmark). Recombinant mycobaterial 65 kDa HSP (HSP65) was kindly provided by Dr. Ruurd van der Zee (Institute of Infectious Diseases and Immunology, Faculty of Veterinary Medicine, Utrecht, The Netherlands). Recombinant HSP60 was prepared as described [6]. Guinea pig myelin basic protein (MBP) was purchased from Sigma (Rehovot, Israel). Two HSP65 peptides were used: Mt176–190 (aa 176–190) EESNTFGLQLELTEG [61] and Mt3 (aa 5–24) AYDEEARRGLERGLNALADA [7]. The Mt176-90 peptide used in this work includes the 180–188 epitope [37]. Two peptides derived from HSP60 were used: p277 (aa 437–460) VLGGGCALLRCPALDSLTPANED and Hu3 (aa 31–50) KFGADARALMLQGVDLLADA. Peptides were synthesized by a standard Fmoc procedure, purified by reverse-phase HPLC and their compositions confirmed by aa analysis. Concanavalin A (Con A) was purchased from Sigma. Incomplete Freund's Adjuvant (IFA) was purchased from Difco.

A monoclonal antibody reactive to rat TCR (clone R73) was purified by us from the hybridoma. Monoclonal antibodies to MHC class-I (MHC-I), MHC class-II RT1.B (MHC-II/ RT1.B), MHC class-II RT1.D (MHC-II/RT1.D), CD28, CD80 and CD86 were purchased from Serotec (Oxford, UK). Purified rabbit anti-human HSP60 polyclonal IgG antibodies were provided by Dr Gabriel Nussbaum (Department of Immunology, The Weizmann Institute of Science, Israel).

### T-cell lines and clones

T-cell lines were raised and expanded as described [38]. Three Lewis rat T-cell lines were used in our experiments: Anti-HSP60, raised against recombinant human HSP60 (human HSP60 is 97% identical to rat HSP60 at the aa level, data not shown); Anti-p277, raised against the p277 peptide of human HSP60 (96% identical its rat counterpart at the aa level, data not shown) and Anti-MBP, raised against guinea pig MBP. For ergotypic stimulation, we used the A2b T-cell clone, specific for the 180–188 epitope of HSP65 [37]; similar results were obtained when other rat T-cell clones were used as targets (data not shown). A2b expresses MHC-I molecules constitutively and CD80, CD86 [62] and MHC-II molecules (not shown) upon activation, and can present peptide epitopes to T cells [63]. Activated A2b cells were used on day 3 of their stimulation, and resting A2b cells were used on day 14–16 of their rest cycle, unless stated otherwise.

### DNA and peptide vaccination

The vectors containing the full-length cDNA of the human *hsp60* gene (pHSP60) or the cDNA corresponding to aa 1–140 (pI) or aa 130–260 (pII) have been previously described [6], [7]. The vector coding for mycobacterial HSP65 (pHSP65) was kindly provided by Dr. Douglas Lowrie (Medical Research Council, London, UK) [30]. The empty vector pcDNA3 was used as a DNA vaccination control.

Plasmid DNA was prepared in large scale and injected after pretreatment with cardiotoxin (Sigma) as previously described [36]. Briefly, rats were vaccinated in the quadriceps three times (on days −40, −26 −12 relative to AA induction) with 150 µg of pcDNA3, pHSP65 or pHSP60. Endotoxin levels were checked by the *Limulus* amoebocyte lysate assay and found always to be under acceptable levels for *in vivo* use (less than 0.02 EU/µg DNA).

Female Lewis rats were immunized intraperitoneally (ip) with a single dose of 100 µg of peptide emulsified in IFA. AA was induced 12 days after the completion of vaccination with DNA or peptide.

### AA Induction and Assessment

AA was induced using heat-killed Mt strain H37Ra (Difco) suspended in IFA, as described [6]. The day of AA induction was designated as day 0. Disease severity was assessed by direct observation of all 4 limbs in each animal. A relative score between 0 and 4 was assigned to each limb, based on the degree of joint inflammation, redness and deformity; thus the maximum possible score for an individual animal was 16. Arthritis was also quantified by measuring hind limb diameter with a caliper. Measurements were taken on the day of the induction of AA and 26 days later, at the peak of AA [6]; the results are presented as the mean±SEM of the difference between the values for hind limb diameter taken on days 0 and 26.

### Anti-ergotipic T-cell proliferation assay

T-cell lines or lymph node cells (LNC, prepared from inguinal and popliteal lymph nodes) were cultured in quadruplicates, 2.5×10^5^ per well, in round-bottom microtiter wells (Nunc, Roskilde, Denmark). Activated or resting A2b stimulator cells were irradiated (5000 R) and added to the test cultures in 2-fold dilutions, starting from 10^5^ cells per well, with no other APC. Con A (1.25 µg/ml) was used as a positive control for T-cell proliferation, and in some experiments the cells were activated with immobilized anti-TCR antibodies as described [64]. Monoclonal antibodies, 10 µg/ml, were added where indicated to test for MHC restrictions or co-stimulation requirements of the anti-ergotypic T cells. Cultures were incubated for 72 hr at 37°C in 7% CO_2_, and pulsed for the last 16 hr with 1 μCi/well of [methyl-^3^H]-thymidine (Amersham, Buckinghamshire, UK). The cultures were harvested and cpm were determined using a beta counter. The ΔCPM was computed as the difference between the mean cpm of wells containing activated or resting A2b stimulator cells to control wells cultured with medium alone.

### Cytokine assays

Supernatants were collected after 72 hr of stimulation with test antigens or stimulator cells. Pharmingen's OPTEIA IL-10, IL-4 and IFNγ kits (Pharmingen, San Diego, USA) and the TGFβ1 E_max_® ImmunoAssay System (Promega, Madison, USA) were used to quantify cytokine release to culture supernatants, as previously described [6]. The lower limits of detection for the experiments described in this paper were 15 pg/ml for TGFβ1, IL-10, IL-4 and IFNγ.

### Western blotting

Cell lysates of resting or activated T cells were prepared by treatment for 15 minutes in the following lysis buffer: NP40 1%, NaCl 0.9%, Tris 50 mM, EDTA 1 mM, PMSF 0.4 mM, pepstatin A 4 µg/ml, leupeptin 4 µg/ml and aprotinin 4 µg/ml. The lysates were centrifuged for 15 min at 14000 rpm and the protein concentration in the supernatant was determined using a BCA protein assay kit (Pierce, Rockford, IL, USA). The lysates were subjected to PAGE-SDS using a mini-gel apparatus (Bio-Rad Laboratories, Hercules, CA); 100 µg of each sample were loaded per well. Two identical gels were run each time in parallel: one gel was stained with Coomassie Brilliant Blue R-250 according to the manufacturer's protocol (Bio-Rad) and the other was electro-transferred to nitrocellulose membranes (Schleicher and Schuell, Dassel, Germany).

The nitrocellulose membranes were washed with PBS and then blocked for 1 hr with 2% bovine serum albumin (Sigma), 2.5% milk powder (Bio-Rad), Tris (Sigma) pH 7.5 10 mM, NaCl 150 mM and 0.02% thimerosal (Sigma). After washing with PBS/Tween 20 (PBST; 0.02%, Sigma), the membranes were incubated in blocking solution for 2 hr with HSP60-specific polyclonal antibodies. The membranes were washed with PBST and incubated with a peroxidase-conjugated goat anti-rabbit IgG (Jackson Immuno-Research, West Grove, PA) at a 1/10000 dilution in blocking solution for 1 hr. Finally, the membranes were developed using the Western Blotting Luminol Reagent (Santa Cruz Biotechnology Inc., Santa Cruz, California, USA), exposed to X-ray film and quantified using the NIH Image 1.63 program (National Institutes of Health, USA). Size was determined using pre-stained broad-range protein standard markers (Bio-Rad).

### Adoptive transfer of anti-ergotypic T cells

Anti-p277 or Anti-MBP T cells were activated for 3 days in culture. Blast cells were isolated using a LymphoPrep gradient (Nycomed, Oslo, Norway), washed, and 5×10^6^ cells per rat were injected ip. Three days later, AA was induced.

### Statistical significance

The InStat 2.01 program was used for statistical analysis. Student's t-test and the Mann-Whitney test were carried out to assay significant differences between the different experimental groups.
